# Supplementary material for: WordCommentsAnalyzer: A windows software tool for qualitative research
Source: F1000Res. 2018 Sep 4;7:536. Originally published 2018 May 3. [Version 2] doi: 10.12688/f1000research.14819.2 (PMC6137411; doi:10.12688/f1000research.14819.2)
Supplement: Supplementary file 3 [file f1000research-7-17614-s0002.tgz › b2a79496-4ada-47c4-9682-a99ce867836d.zip!/Eight Iranian Health Journals/Archives of Rehabilitation_17.docx]

© Archives of Rehabilitation. This work is licensed under CC BY-NC 4.0 (https://creativecommons.org/licenses/by-nc/4.0/).

Volume & Issue: دوره 15 - شماره 1

Title: بررسی اثربخشی آموزش هوش هیجانی بر حرمت خود دانش‌‌آموزان پسر ناشنوا*

http://rehabilitationj.uswr.ac.ir/article-1-1311-fa.html

هدف: هدف این پژوهش بررسی اثربخشی آموزش هوش هیجانی بر حرمت خود دانش‌آموزان ناشنوای شهرستان‌های تهران بود. روش بررسی: پژوهش حاضر، یک مطالعه آزمایشی با طرح پیش آزمون- پس آزمون و گروه کنترل بود. جامعه آماری این پژوهش را دانش‌آموزان پسر ناشنوای دوم راهنمایی شهرستان‌های تهران تشکیل دادند. برای انتخاب آزمودنی‌ها از روش نمونه‌گیری تصادفی خوشه‌ای استفاده شد، به طوری که در این پژوهش 40 دانش‌آموز شرکت داشتند. آزمودنی‌ها به صورت تصادفی به دو گروه 20 نفری تقسیم شدند (یک گروه آزمایش و یک گروه کنترل). گروه آزمایشی، آموزش هوش هیجانی را در 12 جلسه دریافت کردند در حالی که به گروه کنترل این آموزش ارائه نشد. ابزارهای استفاده شده در این پژوهش آزمون هوشی وکسلر کودکان و پرسشنامه حرمت خود کوپر اسمیت بود. اطلاعات جمع‌آوری شده با استفاده از تحلیل کوواریانس چندمتغیری تحلیل شد. یافته‌ها: نتایج نشان داد که بعد از مداخله، میانگین نمرات حرمت خود در گروه آزمایش به طور معناداری بیشتر از گروه کنترل بود (P<0/05). همچنین میانگین نمرات گروه آزمایش در خرده مقیاس‌های حرمت خود خویشتن، حرمت خود اجتماعی، حرمت خود خانواده و حرمت خود تحصیلی به طور معناداری افزایش یافت (P<0/05). نتیجه‌گیری: برنامه آموزشی هوش هیجانی باعث بهبود حرمت خود، حرمت خود خویشتن، حرمت خود اجتماعی، حرمت خود خانواده و حرمت خود تحصیلی دانش‌آموزان ناشنوا شد. بنابراین برنامه‌ریزی برای ارائه آموزش هوش هیجانی به این دانش‌آموزان اهمیت ویژه‌ای دارد.

Title: بررسی قابلیت یادگیری سازمانی و موانع و تسهیل کننده های آن از دیدگاه کارکنان مراکز توانبخشی بهزیستی شهر تهران در سال 1391

http://rehabilitationj.uswr.ac.ir/article-1-1239-fa.html

مقدمه: هدف از انجام این پژوهش تعیین قابلیت یادگیری سازمانی و موانع و تسهیل کننده های آن از دیدگاه کارکنان مراکز توانبخشی بهزیستی شهر تهران در سال 1391بود. روش بررسی: این پژوهش از نوع توصیفی – تحلیلی است که به روش مقطعی اجرا شده است. جامعه آماری شامل کارمندان شاغل در مراکز توان بخشی سازمان بهزیستی شهر تهران در سال 1391 بود. به روش نمونه گیری تصادفی ساده از مراکز با حجم نمونه 278 نفر محاسبه شد. برای جمع آوری دادهها از پرسشنامه محقق ساخته یادگیری سازمانی و پرسشنامه محقق ساخته موانع یادگیری و پرسشنامه محقق ساخته تسهیل کننده های یادگیری سازمانی استفاده شد. یافته‌ها: یافته ها حاکی از بالا بودن قابلیت یادگیری سازمان در کارکنان مراکز توانبخشی بودند (SD=41/5 ,M=2/156) علاوه بر این بین موانع یادگیری سازمانی و قابلیت یادگیری سازمانی رابطه معکوس و معنی داری نیز وجود داشت (P&le0.01، ضریب گاما=6/0-) ولی بین تسهیل کننده های یادگیری سازمانی و قابلیت یادگیری سازمانی رابطه معناداری یافت نگردید (P>0/05، ضریب گاما=16/0-) نتیجه‌گیری: نتایج حاکی از قابلیت بالای یادگیری سازمانی در کارکنان مراکز توانبخشی بهزیستی شهر تهران می باشند. از آنجا که تحقیق نشان داد مهمترین مانع یادگیری سازمانی فشار کاری روزانه کارکنان و مهمترین تسهیل کننده یادگیری سازمانی شناخت نقاط قوت و ضعف کارکنان در یادگیری در بین کارکنان مراکز توانبخشی است، لذا به منظور ارتقا بهره وری سازمانی توجه کافی به این موانع و برنامه ریزی جهت حذف یا کاهش موانع یادگیری سازمانی و افزایش تسهیل کننده های یادگیری سازمانی کارکنان مراکز توانبخشی پیشنهاد می شود.

Title: طراحی فرم مکمل مقیاس عملکرد کاری کانادایی: تجزیه و تحلیل آیتم و پیشنهاداتی برای پالایش

http://rehabilitationj.uswr.ac.ir/article-1-1345-fa.html

هدف: هدف این مطالعه، تعیین روایی افزایشی فرم تجربی در مقایسه با فرم COPM به تنهایی، در تعیین مشکلات عملکرد کاری مادران دارای کودک فلج مغزی بود. روش بررسی: پژوهش حاضر، مطالعه ی دو مرحله ایِ پالایش آیتم، برای فرم تجربی مکمل مقیاس COPM بود. در مرحله اول، طراحی فرم تجربی و انجام امکان تکرارپذیری آن و در مرحله دوم پالایش آیتم ها صورت گرفت. برای بررسی امکان تکرار پذیری، فرم تجربی در اختیار 30 نفر از مادران کودکان فلج مغزی، و به منظور پالایش آیتمهای فرم تجربی، در ابتدا نسخه فارسی COPM و در ادامه فرم تجربی در اختیار 21 نفر از مادران مراجعه کننده به کلینیکهای کاردرمانی شهر تهران قرار گرفت یافته‌ها: در مرحله اول، ضریب تکرار پذیری نسبی برای همه خرده مقیاس های فرم تجربی، بالای 0/7 بود. در مرحله پالایش آیتم، در زیر بخشهای مدیریت امور خانه، تفریح بی سر و صدا، تفریح پر جنب و جوش و معاشرت در فرم COPM به تنهایی با فرم COPM بهمراه فرم تجربی تفاوت معنی داری دیده شد (0/01>P). نتیجه‌گیری: فرم تجربی کمک می کند تا مشکلات عمکرد کاری مهمتر و بیشتری از مادران کودکان فلج مغزی مشخص گردد. لذا استفاده از فرم تجربی، برای شناخت بهتر تغییرات اکوپیشنهای مادران کودکان فلج مغزی توصیه می شود.

Title: آیا می‌توان از کفش ناپایدار به عنوان کفش تمرینی ایمن در تمرینات بازتوانی و توانبخشی استفاده نمود؟

http://rehabilitationj.uswr.ac.ir/article-1-1396-fa.html

هدف:کفش ناپایدار به عنوان عاملی ایمن برای افراد سالم و ابزاری جهت کاهش درد در افراد استئوآرتریتی حین راه رفتن پیشنهاد شده است.هدف از تحقیق حاضر،تأثیر کفش ناپایدار بر متغیرهای بیومکانیکی مرتبط با آسیب(سودمندی آن به عنوان کفش توانبخشی ایمن)هنگام دویدن در افراد سالم بود. روش بررسی: 25 نفر آزمودنی مرد جوان سالم (میانگین سنی 27/2±21 سال) به صورت در دسترس در این آزمون شرکت کرده و در شرایط پابرهنه،با کفش ناپایدار و کفش کنترل روی صفحه نیروسنج دویدند.متغیرهای توان مفصلی مثبت و منفی،حداکثر نیروی عمودی فعال و غیرفعال،نیروهای قدامی و خلفی،سرعت بارگذاری و ضربه نیروهای عمودی غیرفعال وخلفی طی همزمان‌سازی دوربین ویدئویی و صفحه نیروسنج ثبت و با استفاده از نرم افزار MATLAB محاسبه گردیدند.آزمون فرضیات با روش تحلیل واریانس با داده‌های تکراری در نرم افزار SPSS(نسخه20) انجام شد (P<0.05). یافته‌ها:توان منفی مچ‌پا در کفش ناپایدار در مقایسه با کفش کنترل و پای‌برهنه به ترتیب 49% و 35%به طور معناداری کاهش یافت (P<0/05 ,P=0/02). کفش ناپایدار توان مثبت مچ‌پا را در مقایسه با پای برهنه 23% کاهش معنادار داد (P=0/01). متغیرهای حداکثر نیروی عمودی غیرفعال وسرعت بارگذاری آن،حداکثر نیروی خلفی وضربه ناشی از آن هنگام دویدن باکفش ناپایدار در مقایسه باکفش کنترل به طورمعناداری افزایش یافت (P<0/05). نتیجه‌گیری: کفش ناپایدار برخی از ریسک فاکتورهای آسیبی دویدن شامل حداکثر نیروی عمودی غیرفعال وسرعت بارگذاری آن،حداکثرنیروی خلفی وضربه حاصل از آن را افزایش داد.این کفش‌ باکاهش توان منفی مچ‌پا نسبت به کفش کنترل،منجر به کاهش ظرفیت جذبی وکنترلی عضلات ساق و پایداری فعال این مفصل می‌شود.با وجود این،با افزایش توان مثبت مچ‌پا در مقایسه با پای برهنه وکاهش نیروی قدامی،می‌تواند ظرفیت پیشروی و تولید نیرو را در این مفصل بهبود بخشد.

Title: عملکرد دانش‌آموزان دیرآموز اول ابتدایی در مقیاسهای هوشی وکسلر، لایتر و گودیناف

http://rehabilitationj.uswr.ac.ir/article-1-1287-fa.html

هدف: این پژوهش، با هدف بررسی عملکرد دانش‌آموزان دیرآموز اول ابتدایی در مقیاس‌های هوشی وکسلر، لایتر و گودیناف به مرحله اجرا درآمد. روش بررسی: این پژوهش از نوع مقایسه‌ای است. جامعه آماری شامل همه دانش‌آموزان دیرآموز اول ابتدایی شهر اصفهان بود که از میان‌ آن‌ها، تعداد 50 دانشآموز (25 دختر و 25 پسر) با روش نمونه‌گیری تصادفی ساده انتخاب شدند. از مقیاس تجدید‌ نظر شده هوش وکسلر کودکان، مقیاس عملکردی بین‌المللی لایتر و آزمون نقاشی آدمک گودیناف،‌ جهت سنجش هوش کلامی و غیرکلامی دانش‌آموزان استفاده شد. یافته‌ها: با استفاده از آزمون‌‌های آماری ضریب همبستگی پیرسون و تی مستقل تحلیل شدند. یافته‌ها: نتایج نشان داد که دانش‌آموزان دیرآموز، در خردهمقیاس‌های وکسلر و لایتر دارای عملکردی مشابه بودند. مقیاس لایتر با وکسلر (P<0/05) و گودیناف (P<0/01) رابطه مثبت معنی‌داری دارد. همچنین، بین دانش‌آموزان دختر و پسر از نظر مقیاس‌های هوشی وکسلر، لایتر و گودیناف تفاوت معنی‌داری وجود ندارد (P>0/05). نتیجه‌گیری: ارتباط بین مقیاس‌ هوشی لایتر با وکسلر و گودیناف، نیز تأییدی بر صحت ارزیابی است. عملکرد دیرآموزان دختر و پسر اول ابتدایی در سه مقیاس‌‌ هوشی مشابه بود.

Title: بررسی میزان شیوع اختلالات رفتاری در بین دانش‌آموزان دوره متوسطه در استان همدان

http://rehabilitationj.uswr.ac.ir/article-1-1415-fa.html

هدف: اختلالات رفتاری طیف وسیعی از مشکلات دوران کودکی و نوجوانی را تشکیل می‌دهد که شیوع آن در مناطق مختلف ایران و جهان متفاوت است و آگاهی از آن، لازمه پیشگیری از آن و درمان و توانبخشی مبتلایان است. پژوهش حاضر به بررسی میزان شیوع اختلالات رفتاری در دانش‌آموزان دوره متوسطه استان همدان پرداخته است. روش بررسی: این پژوهش توصیفی، از نوع زمینه‌یابی مقطعی می‌باشد. جامعه آماری آن تمامی دانش‌آموزان دوره متوسطه استان همدان در سال تحصیلی ۹۲-۹۱ بود و حجم نمونه ۵۰۰ نفر دانش‌آموز دختر و پسر بود که با استفاده از جدول کرجسی و مورگان و به شیوه نمونه‌گیری خوشه‌ای چندمرحله‌ای بر اساس منطقه و جنسیت، انتخاب شدند. برای جمع‌آوری اطلاعات از پرسشنامه مبتنی بر تجربه آخنباخ فرمYSR استفاده شد و برای تحلیل داده‌ها از آزمون فریدمن و تی مستقل استفاده شد. یافته‌ها: نتایج نشان داد که میزان شیوع اختلالات رفتاری در بین دانش‌آموزان استان همدان ۱۰ درصد است. میزان شیوع در بین دختران بیشتر از پسران بوده و بیشترین اختلال مربوط به مشکلات گوشه گیری/اضطراب و مشکلات توجه و کمترین اختلال مربوط به مشکلات و شکایات جسمانی می‌باشد. نتیجه‌گیری: میزان شیوع اختلالات رفتاری در استان همدان مشابه با سایر پژوهش‌های انجام شده بوده، اما میزان شیوع در جنسیت با سایر تحقیقات متفاوت است.

Title: مقایسه تأثیرات تمرین درمانی و روش پیلاتس بر عملکرد و حس عمقی بیماران مبتلا به استئوآرتریت زانو: یک کارآزمایی کنترل شده تصادفی

http://rehabilitationj.uswr.ac.ir/article-1-1310-fa.html

هدف: استئوآرتریت زانو از جمله شایع ترین اختلالات عضلانی اسکلتی محسوب می شود که باعث تخریب مفصل و متعاقب آن کاهش سطح عملکرد و نقص حس عمقی می شود. هدف از این مطالعه بررسی تأثیر تمرینات پیلاتس بر استئوآرتریت زانو بود. روش بررسی: تعداد 41 مرد بیمار مبتلا به استئوآرتریت زانو به صورت تصادفی در سه گروه تحت درمان با تمرینات پیلاتس، حرکت درمانی معمولی و کنترل قرار گرفتند. حس عمقی، سطح عملکرد، و ارزیابی شرایط بیماری به ترتیب توسط سیستم بایودکس، اندازه گیری مدت زمان انجام چهار فعالیت عملکردی و پرسشنامه Lequesne پیش از اعمال مداخله و پس از 8 هفته مورد بررسی قرار گرفتند. جهت تجزیه و تحلیل داده های آماری از آمار توصیفی، آزمون تحلیل واریانس (ANOVA) و آزمون تعقیبی شفه در سطح معنی داری 5 درصد استفاده شد. یافته‌ها: مشخصات دموگرافیک آزمودنی ها در مورد سن و مدت ابتلای به عارضه به صورت (میانگین±انحراف معیار: سن 9/8±1/52 سال، مدت ابتلا 2/11±3/23 ماه) بود.سطح عملکرد و میزان خطای بازسازی زاویه هدف در هر دو گروه تجربی در مقایسه با گروه کنترل به طور معناداری (0/001>P) بهبود یافت، اما تفاوت بین گروههای تحت درمان معنادار نبود (0/05>P). شاخص Lequesne در هر دو گروه تمرینات پیلاتس و حرکت درمانی نسبت به گروه کنترل به طور معناداری (0/001>P) بهبود یافت، با این حال اثرات روش پیلاتس در بهبود این شاخص به طور معناداری (0/05>P) بیشتر از روش حرکت درمانی بود. نتیجه‌گیری: تکنیک درمانی پیلاتس می تواند باعث بهبود حس عمقی و ارتقای سطح عملکرد بیماران مبتلا به استئوآرتریت زانو شود. مطالعات بیشتری جهت پی بردن به ساز و کار این نوع تمرینات نیاز است.

Title: تاثیر نوارپیچی مچ پا به روش مولیگان بر تعادل پویا ورزشکاران با و بدون ناپایداری مزمن مچ پا

http://rehabilitationj.uswr.ac.ir/article-1-1280-fa.html

هدف: مفصل مچ پا بخش آناتومیکی است که در معرض بیشترین آسیب در بین ورزشکاران قرار دارد. ناپایداری مچ پا مسئول %25 همه زمان‌هایی است که ورزشکاران به‌دلیل آسیب نمی‌توانند در فعالیت ورزشی شرکت کنند. کارایی بالینی نوارپیچی در ورزشکاران با ناپایداری مچ پا همچنان در ابهام است. بنابراین هدف از این پژوهش، بررسی تاثیر نوارپیچی مچ پا به‌روش مولیگان بر تعادل پویای ورزشکاران با ناپایداری مزمن مچ پا و ورزشکاران سالم است. روش بررسی: 32 ورزشکار شامل 16 فرد با ناپایداری مزمن مچ پا (6 زن و 10 مرد، سن؛ 0/3±5/23 سال، قد؛ 3/10±4/175 سانتیمتر، وزن؛ 5/14±60/73 کیلوگرم) با نمره پرسشنامه شاخص‌ ناتوانی پا و مچ پا %62/8±5/74 و نمره پرسشنامه شاخص ورزشی ناتوانی پا و مچ پا %68/7±5/63 و 16 فرد سالم (6 زن و 10 مرد، سن؛ 1/7±81/22 سال، قد؛ 26/12±6/173 سانتیمتر، وزن؛ 4/11±4/66 کیلوگرم) با نمره پرسشنامه شاخص‌ ناتوانی پا و مچ پا %100 و نمره پرسشنامه شاخص‌ ورزشی ناتوانی پا و مچ پا %100 در این مطالعه شرکت کردند. تعادل پویا بوسیله آزمون تعادلی ستاره در جهات داخلی، قدامی–داخلی و خلفی–داخلی قبل و پس از نواربندی به‌شیوه مولیگان، ارزیابی شد. برای تجزیه و تحلیل آماری از آزمون تی مستقل و همبسته استفاده شد. یافته‌ها: تعادل پویا در گروه سالم در مقایسه با گروه آسیب‌دیده به‌طور معناداری بهتر بود (0/05&leP). کاربرد نوارپیچی باعث بهبود معنادار تعادل پویا (دستیابی در جهات داخلی، قدامی–داخلی و خلفی–داخلی) در دو گروه شد (0/05&leP) (بجز دستیابی در جهت قدامی– داخلی در گروه سالم) (0/05). نتیجه‌گیری: بنابراین، به‌نظر می‌رسد نوارپیچی مچ پا به روش مولیگان می‌تواند تعادل پویا را در ورزشکاران با و بدون ناپایداری مزمن مچ پا بهبود بخشد.

Title: بررسی رشد حرکتی شیرخواران 2-0 ساله شهر همدان براساس آزمون پی بادی

http://rehabilitationj.uswr.ac.ir/article-1-1389-fa.html

هدف: حرکت یکی از جنبه های رشدی فرد است که از بدو تولد تا آخر عمر همراه او بوده و مهمترین عامل برای رسیدن فرد به اهداف و تامین نیازهای خویش است. بررسی دقیق مهارت های حرکتی درشت و ظریف در شیرخوارگی، تشخیص و درمان زودهنگام اختلالات حرکتی را آسانتر می نماید. لذا پژوهش حاضر با هدف بررسی رشد حرکتی شیرخواران 2-0 ساله شهر همدان انجام شد. روش بررسی: مطالعه حاضر از نوع مقطعی و توصیفی تحلیلی بوده که 124 شیرخوار به روش نمونه گیری خوشه ای تصادفی از بین مراکز بهداشت شهر همدان تحت سنجش قرار گرفتند. ابزار جمع آوری اطلاعات آزمون رشدی حرکتی پی بادی (PDMS) بود. به منظور تجزیه و تحلیل داده ها از نرم افزار آماری SPSS-16 استفاده شد. یافته‌ها: نتایج مطالعه نشان داد اختلاف معناداری از نظر متغیرهای جنس، نوع زایمان، مرتبه زایمان، سابقه مصرف سیگار پدر و سابقه مصرف سیگار مادر در رشد حرکتی درشت و ظریف وجود ندارد. براساس یافته ها با افزایش سن، مهارت حرکتی درشت، ظریف و کل، همچنین و بهره رشد حرکتی درشت، ظریف و کل افزایش می یابد (0/001>P). نتیجه‌گیری: میانگین سن حرکتی و همچنین بهره رشد حرکتی شیرخواران مورد مطالعه در محدوده بهنجار بود، با این حال برنامه های تقویت حرکتی برای تسهیل روند رشد حرکتی شیرخواران پیشنهاد می گردد.

Title: مشکلات جداسازی اصوات گفتاری همزمان در کودکان کم شنوا

http://rehabilitationj.uswr.ac.ir/article-1-1347-fa.html

هدف: مطالعه حال حاضر، در زمره پژوهشهای بنیادین انجام گرفته برای بررسی توانایی جداسازی اصوات گفتاری همزمان در کودکان کم شنوا می باشد. جداسازی همزمان، یکی از مولّفه های پایه ای رویکرد تجزیه و تحلیل صحنه شنوایی (ASA) بوده و نقش مهمّی در فرآیند درک گفتار، دارا می باشد. در این مطالعه، پتانسیلهای دیررس شنوایی کودکان کم شنوا بررسی شده و با پاسخ های گروه هنجار مقایسه شدند. روش بررسی: در 10 کودک هنجار و 10 کودک کم شنوای 4 تا 6 ساله، پاسخ های دیررس شنوایی یا ALRs (دامنه مجموعه N1-P2) برای دوازده جفت واکه سنتز شده ای که به طور همزمان و به صورت دوگوشی ارائه می شدند، ثبت گردیدند. تفاوت جفت واکه های مورد نظر، در فرکانس پایه آنها بود؛ به نحوی که، فرکانس پایه هر یک از آنها به میزان نیم semitone از دیگری تفاوت داشت. یافته‌ها: مقایسه دامنه مجموعه N1-P2 میان دو گروه هنجار و کم شنوا نشان دهنده وجود اختلاف آماری معنادار در شماری از جفت واکه ها بود (P<0/05). با بررسی دقیقتر نتایج، دامنه مجموعه N1-P2 (که شاخصی از کشف تغییرات واکه ها و بازنمایی تحریکات گفتاری در دستگاه شنوایی مرکزی در شرایط نبود توجّه فعّال شرکت کنندگان می باشد) در گروه کودکان کم شنوا از مقدار کمتری برخوردار بود. نتیجه‌گیری: پژوهش حاضر گویای اختلال جداسازی اصوات گفتاری همزمان در کودکان کم شنوا می باشد. این مطالعه، شواهدی را درباره بروز مشکلاتی در کشف ویژگی های گفتاری در مسیر پایین به بالا (مبتنی بر فرکانس پایه و تفاوت های آن) در کودکان مذکور فراهم می نماید.
